# Characteristics of Mortars with Blast Furnace Slag Powder and Mixed Fine Aggregates Containing Ferronickel-Slag Aggregate

**DOI:** 10.3390/ma14195879

**Published:** 2021-10-08

**Authors:** Sung-Ho Bae, Jae-In Lee, Se-Jin Choi

**Affiliations:** Department of Architectural Engineering, Wonkwang University, Iksan 54538, Korea; caos1344@naver.com (S.-H.B.); wodls103@naver.com (J.-I.L.)

**Keywords:** blast furnace slag powder, steel slag aggregate, compressive strength, carbonation depth, chloride ion penetrability

## Abstract

Recently, interest in environmentally friendly development has increased worldwide, especially in the construction industry. In this study, blast furnace slag powder (BFSP) and mixed steel fine aggregates were applied to cement mortars to reduce the environmental damage caused by the extraction of natural aggregate and to increase the recycling rate of steel by-products in the construction industry. We investigated the fluidity, compressive strength, tensile strength, accelerated carbonation depth, and chloride ion penetration resistance of mortars with steel slag aggregate and their dependence on the presence or absence of BFSP. Because the recycling rate of ferronickel slag is low and causes environmental problems, we considered mortar samples with mixed fine aggregates containing blast furnace slag fine aggregate (BSA) and ferronickel slag fine aggregate (FSA). The results showed that the 7-day compressive strength of a sample containing both 25% BSA and 25% FSA was nearly 14.8% higher than that of the control sample. This trend is likely due to the high density and angular shape of steel slag particles. The 56-day compressive strength of the sample with BFSP and 50% FSA was approximately 64.9 MPa, which was higher than that of other samples with BFSP. In addition, the chloride ion penetrability test result indicates that the use of BFSP has a greater effect than the use of steel slag aggregate on the chloride ion penetration resistance of mortar. Thus, the substitute rate of steel slag as aggregate can be substantially enhanced if BFSP and steel slag aggregate are used in an appropriate combination.

## 1. Introduction

Recently, the interest in environmentally friendly development has increased worldwide, especially in the construction industry. Because of the depletion of natural aggregate resources and environmental pollution problems, research is being conducted to develop alternative aggregates or use industrial by-products [1,2,3,4]. 

Aggregates make up a significant portion of mortar or concrete volume, and the quality of mortar or concrete depends on the quality of the aggregate. In addition, due to the problem of aggregate shortage, aggregate prices sometimes rise, causing production disruptions in the concrete industry. Among the various industrial by-products, steel slag produced in the form of aggregate from the steel industry can be a good alternative as aggregate for mortar or concrete. In Korea, approximately three million tons of ferronickel slag, a by-product of nickel production, are generated annually [5], and various efforts are being made to increase the recycling rate of ferronickel slag [6,7,8,9]. 

Several lines of research related to ferronickel slag have been reported in the literature [10,11,12,13,14,15,16,17]. Saha et al. [11] reviewed the usability of electric furnace ferronickel slag (FNS) as a fine aggregate and binder, and reported that powdered FNS may exhibit a pozzolanic reaction when used as a cement substitute. Katsiotis et al. [12] reviewed the usability of ferronickel slag as an additive and reported that the concentrations of leaked heavy metals were quite low. You [13] reviewed the characteristics of alkali-activated blended slag mortar using ferronickel slag and reported that the heat of hydration and porosity were lower than those of Portland cement mortar. In addition, Lee et al. [16] evaluated the mechanical properties of concrete using FSA; the compressive strength of the concrete increased with increasing FSA mixing ratio. Additionally, they reported an enhanced compressive strength of concrete when the FSA mixing ratio exceeded 30%. Nuruzzaman et al. [17] investigated the characteristics of high-strength concrete using FSA and reported that the durability of high-strength concrete can be improved with appropriate FSA.

However, the recycling rate of ferronickel slag is still low, and unused ferronickel slag is left unattended in factories or is illegally dumped, causing deterioration of factory operation and polluting the surrounding environment.

If the use of the discarded ferronickel slag as an aggregate can be enhanced, it can have multiple advantages in alleviating the problem of insufficient aggregate for mortar or concrete and preventing environmental pollution caused by steel by-products. Therefore, we applied combinations of blast furnace slag aggregate (BSA) and ferronickel slag aggregate (FSA) to mortar to alleviate the problem of aggregate shortage and to increase the recycling rate of steel slag, including ferronickel slag. In addition, we examined the application of blast furnace slag powder (BFSP) in mortar using the steel slag aggregate. The use of BFSP to reduce greenhouse gas emissions in the cement industry has been rising [18,19,20,21,22,23,24].

There are some previous studies on using FSA and BSA as aggregates [25,26,27,28], but there are no reports on using BFSP with mixed steel slag aggregate containing BSA and FSA. In the concrete industry that consumes a large amount of aggregate, the combined use of mixed steel slag aggregate and BFSP is expected to alleviate the problem of aggregate shortage and to considerably increase the recycling rate of steel by-products that can cause environmental pollution. 

Therefore, in this study, we investigated the fluidity, compressive strength, tensile strength, accelerated carbonation depth, and chloride ion penetration resistance of mortar with mixed steel slag aggregate containing FSA and BSA and their dependence on the presence or absence of BFSP.

## 2. Materials and Experimental Methods

### 2.1. Materials

We used ordinary Portland cement (Specific gravity: 3.15, Blaine: 3430 cm^2^/g) from Asia Co., Korea, and blast furnace slag powder (BFSP) (Specific gravity: 2.93, Blaine: 4210 cm^2^/g) produced by Daehan Slag Co. Ltd., Korea. For this study, we selected the maximum mixing ratio of steel slag aggregate as 50% by referring to the existing literature [9,28], and used natural aggregates for the rest.

**Figure 1:**
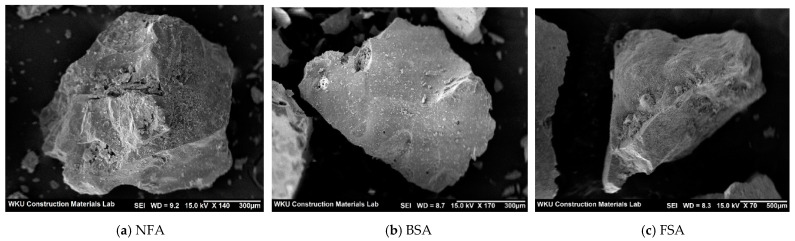
SEM images of NFA, BSA and FSA: (**a**) NFA, (**b**) BSA, (**c**) FSA.

As fine aggregates, a natural fine aggregate (NFA), BSA, and FSA supplied by POSCO, Korea, were used. NFA, BSA, and FSA have specific gravity values of 2.60, 2.81, and 3.05, respectively, and fineness modulus of 2.89, 2.37, and 3.51, respectively. Figure 1 shows images of NFA, BSA and FSA, and Table 1 and Table 2 list the chemical compositions of the cementitious materials and physical properties of the fine aggregates. As shown in the figure, the shapes of BSA and FSA are more irregular and angular than that of NFA.

**Figure 2:**
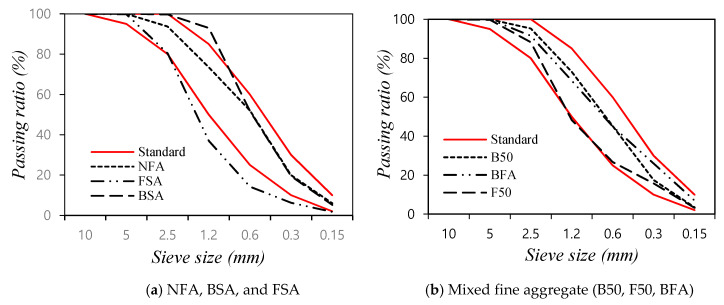
Particle size distribution of fine aggregates used in this study: (**a**) NFA, BSA, and FSA; (**b**) mixed fine aggregate.

Figure 2 shows the particle size distributions of the fine aggregates; the particle size distributions of BSA and FSA were outside the standard range. However, the particle size distributions of mixed fine aggregates (B50, BFA, and F50) were mostly within the standard range.

### 2.2. Mixing Proportions and Specimen Preparation

Table 3 shows the mixing proportions in the cement mortar. The water–binder ratio was fixed at 50%, and the replacement proportion of the steel slag aggregate was set at a maximum of 50%, considering the particle size distribution of the mixed fine aggregate. To examine the characteristics of the mortar with mixed steel slag aggregate in the presence of BFSP, cement mortar samples were prepared using 100% cement (C-B50, C-BFA, and C-F50); BFSP mortar samples were prepared using 40% BFSP and 60% cement (B-B50, B-BFA, and B-F50). The amount of BFSP used was fixed at 40% and no chemical admixture was used.

Mortar samples with mixed fine aggregates were divided into B50 (NFA 50% and BSA 50%), F50 (NFA 50% and FSA 50%), and BFA (NFA 50%, BSA 25%, and FSA 25%) depending on the composition of NFA and steel slag fine aggregate. Furthermore, 50 mm × 50 mm × 50 mm cubic specimens were prepared via molding for compressive strength testing, and 50 mm × 100 mm cylindrical specimens were prepared for split-tensile strength testing. Additionally, 40 mm × 40 mm × 160 mm specimens were prepared for accelerated carbonation testing, and 100 mm × 50 mm specimens were prepared for the chloride ion penetration test. Subsequently, the specimens were demolded after 24 h and cured in a water tank at 20 °C to the required age. The mortar flow and compressive strength were measured according to KS L 5105 [29], and the tensile strength was measured according to KS F 2423 [30]. The presented strength values are the average values of three samples.

**Figure 3:**
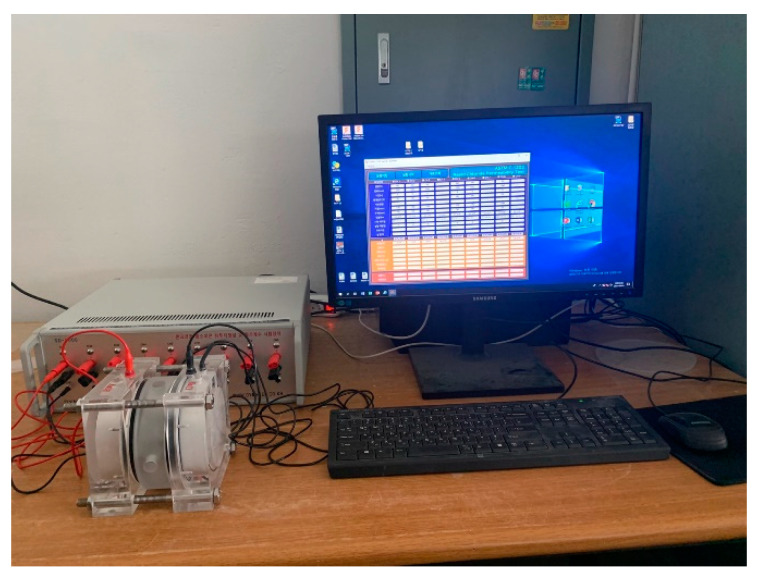
Chloride-ion penetration test.

For carbonation test, the carbonation depth was measured using a phenolphthalein solution after the carbonation process in an accelerated carbonation chamber, according to KS F 2584 [31]. Additionally, the chloride ion penetration test (Figure 3) was performed according to ASTM C 1202 [32]. This test method consists of monitoring the amount of electrical current passed through the samples. A potential difference of 60 V DC is maintained across the ends of the specimen, one of which is immersed in a sodium chloride solution, the other in a sodium hydroxide solution.

## 3. Results and Discussion

### 3.1. Mortar Flow

**Figure 4:**
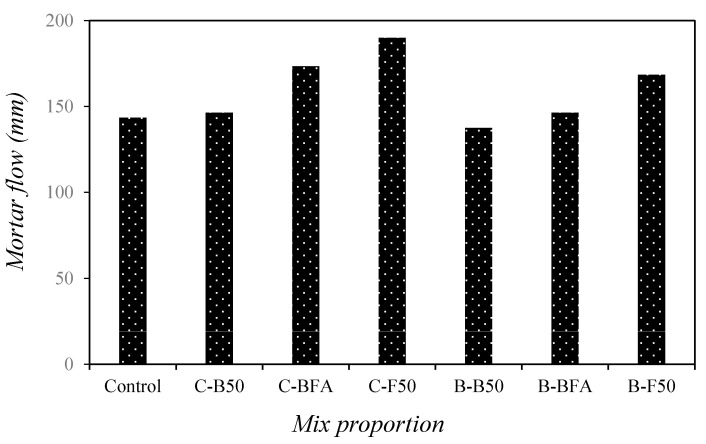
Changes in mortar flow with mixed fine aggregates.

Figure 4 shows changes in the mortar flow with the mixed fine aggregates, including NFA, BSA, and FSA. As can be seen, the flow of the control sample using only NSA was approximately 143 mm. Among the samples using only cement as the binder, the C-F50 sample with 50% FSA showed the highest flow of approximately 174 mm, which is approximately 21% higher than that of the control sample. Additionally, the mortar flow value increased with increasing amount of FSA. Among the samples with 40% BFSP as a cement substitute, the flow value of the B-F50 sample was the highest. The flow value of the B-F50 sample was 168.5 mm, which was about 17.4% higher than that of the control sample.

The flow of the samples with BFSP was approximately 7–18% lower than that of the samples with only cement. In addition, even in the samples with BFSP, the fluidity of the mortar increased with increasing amount of FSA, probably because of the low absorption rate and the glassy properties of FSA [33].

### 3.2. Compressive Strength

**Figure 5:**
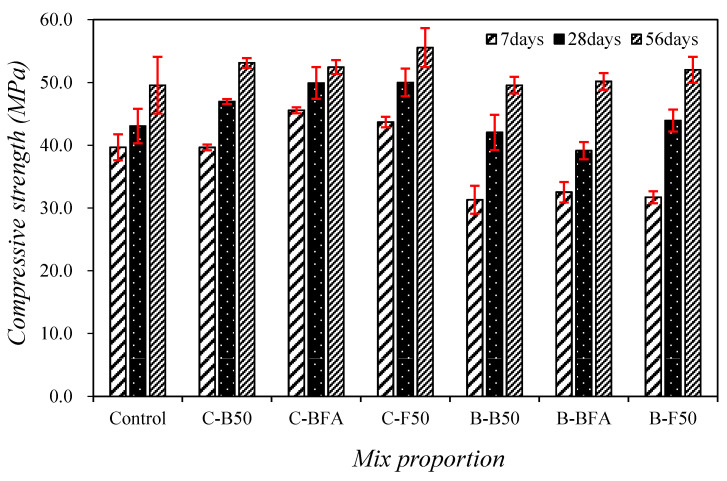
Changes in compressive strength of the mortar with mixed fine aggregate.

Figure 5 shows the changes in compressive strength of the mortar with mixed fine aggregate.

The 7-day compressive strength of the control mixture was approximately 39.7 MPa, and those of the mixtures using mixed fine aggregate and only cement as a binder were similar to or higher than that of the control mixture. Particularly, the compressive strength of the C-BFA sample containing 25% BSA and FSA was approximately 45.6 MPa, nearly 14.8% higher than that of the control sample. In this study, the compressive strength of the mortar with steel slag aggregate was improved due to the high density of steel slag and improved adhesion strength [9,11,12,21]. This trend was greater in the samples using FSA. However, the 7-day compressive strength of the specimen with BFSP was approximately 22% lower than that of the control specimen. This trend is likely because the amount of cement was relatively small and the curing period was short, so the effect of latent hydraulic reaction of BFSP and steel slag aggregate was relatively low.

After 28 days, compressive strength of the control mixture was approximately 43.0 MPa; using only cement as the binder, the compressive strengths of the mixtures with the steel slag aggregate were approximately 9–16% higher than that of the control mixture. Additionally, when using BFSP, the compressive strengths of the samples with steel slag aggregate increased continuously, and the 28-day compressive strengths of the mixtures using the steel slag aggregate were approximately 39.1–43.9 MPa. In particular, the 28-day compressive strength of B-F50 using BFSP and 50% FSA was approximately 43.9 MPa, which is a significant improvement compared to the control sample.

Even after aging for 56 days, the compressive strengths increased continuously for all mixtures using mixed fine aggregate, the compressive strength of the sample using BFSP and steel slag aggregate was about 49.6–52.0 MPa, which was slightly higher than that of the control sample.

### 3.3. Tensile Strength

**Figure 6:**
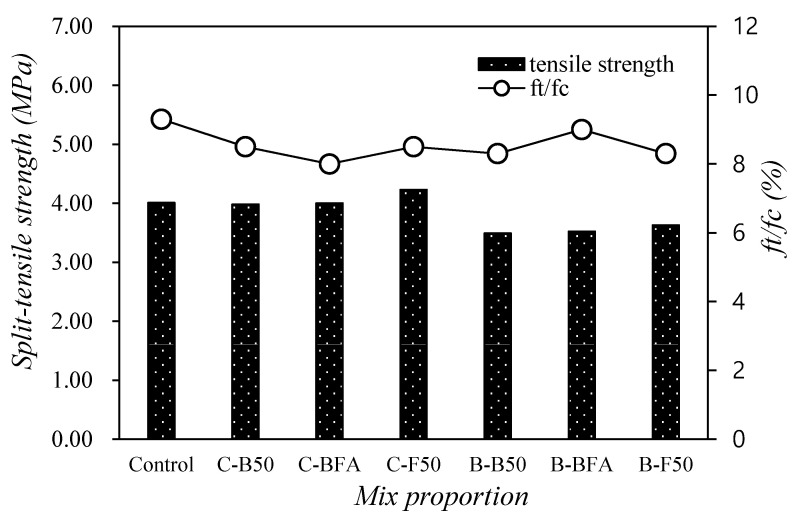
Tensile strength of mortar with mixed fine aggregates at 28 days.

Figure 6 shows the change in the split-tensile strength of mortar with mixed fine aggregates at 28 days. The tensile strength of the control mixture was approximately 4.0 MPa. Among the mixtures using only cement as the binder, the tensile strengths of C-B50 and C-BFA were similar to that of the control mixture. Particularly, the tensile strength of the C-F50 sample with 50% FSA was approximately 4.23 MPa, 5.4% higher than that of the control sample. 

When 40% of the BFSP was used as the binder, the tensile strength of the B-F50 sample using 50% FSA was relatively high. In the case of tensile strength, unlike the compressive strength trend, the tensile strength of the sample using BFSP was around 3.4 to 3.6 MPa, which was 9.4 to 12.9% lower than that of the control sample. It seems necessary to evaluate the change in tensile strength across longer timespans.

The ratio of tensile strength to compressive strength was approximately 8–9% regardless of the content of steel slag fine aggregate.

### 3.4. Accelerated Carbonation Depth

**Figure 7:**
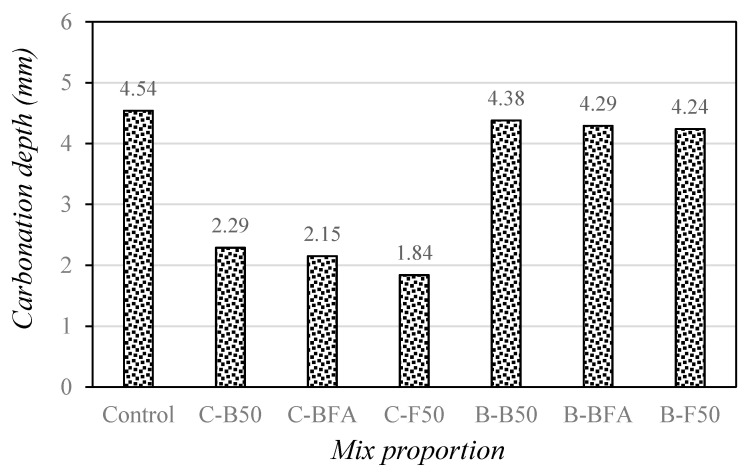
Changes in carbonation depth of mortar with mixed fine aggregates at 28 days.

Figure 7 shows the change in the carbonation depth of mortar with mixed fine aggregate after 28 days of accelerated carbonation. 

As shown in the figure, the carbonation depth of the control sample was the highest at approximately 4.54 mm, and the carbonation depth of the C-F50 sample with 50% FSA was the lowest (1.84 mm). For the mixture using steel slag aggregates and cement, the carbonation depth was approximately 50–59% lower than that of the control sample. Therefore, it is estimated that the carbonation resistance of cement mortar can be effectively improved by using mixed steel slag aggregate.

For the mixtures with 40% BFSP as a cement substitute, the carbonation depth was larger than that of the samples using only cement due to the reduced alkalinity resulting from the decrease in the cement content. However, the accelerated carbonation depth of all samples using BFSP and steel slag aggregate is around 4.24–4.38 mm, which is approximately 3.5–6.6% lower than that of the control sample. This shows that the carbonation resistance of the mortars can be improved compared to the control sample by using the mixed steel slag aggregate, although the cement content was 40% lower than that of the control sample.

In particular, the carbonation depth of the B-F50 sample using 50% FSA was lower than that of the B-B50 sample using 50% BSA, indicating that the use of FSA was more effective in improving the strength and carbonation resistance of mortar than BSA.

### 3.5. Chloride Ion Penetrability

**Figure 8:**
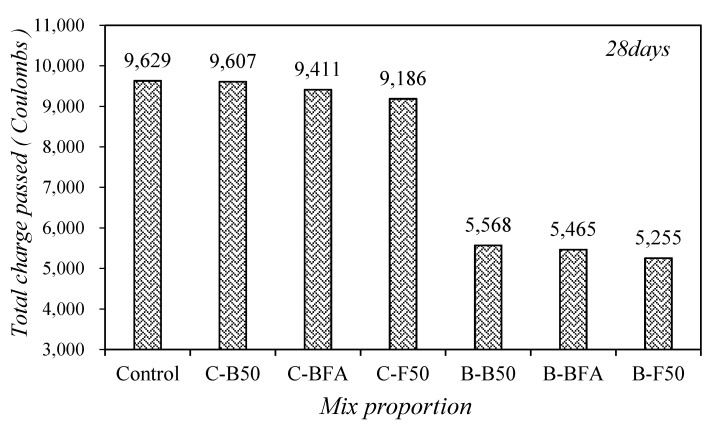
Changes in chloride ion penetrability of mortar with mixed fine aggregates.

Figure 8 shows the change in the chloride ion penetrability of mortar with mixed fine aggregates. The total charge passing through the control mixture was the highest at approximately 9629 C, and the total charge passing through the C-B50 sample using 50% BSA was slightly lower than that through the control sample, and the total charge passing through the samples decreased as the amount of FSA increased.

Although existing literature reports that the chloride-ion permeability of concrete was significantly improved when mixed steel slag aggregate was used [27], the decrease in chloride-ion permeability of the sample using steel slag aggregate was not significant in this study.

By replacing 40% of the BFSP with a cement substitute, the total charge passing through each sample was approximately 5255–5568 C, which is roughly 42–45% lower than that for the control sample, and the B-F50 samples with BFSP and 50% FSA showed the maximum resistance to chloride ion penetration. In addition, in the case of the samples using the steel slag aggregate, the total charge passing through the samples using the steel slag aggregate and BFSP together was about 41.9–42.7% lower than that of the sample without the BFSP.

Therefore, in this study, it was shown that chloride ion penetration resistance was greatly improved when steel slag aggregate and BFSP were used together, rather than using only mixed steel slag aggregate.

## 4. Conclusions

The conclusions of this study are as follows:

1. The 7-days compressive strength of the C-BFA sample containing 25% BSA and FSA was approximately 45.6 MPa, approximately 14.8% higher than that of the control sample. Moreover, the 28-day compressive strength of B-F50 using 40% BFSP and 50% FSA was approximately 43.9 MPa, which is higher than that of the control sample.

2. The tensile strength of the C-F50 sample with 50% FSA was approximately 4.23 MPa, nearly 5.4% higher than that of the control sample. 

3. For the mixture using steel slag aggregates and cement, the carbonation depth was approximately 50–59% lower than that of the control sample. Therefore, it is estimated that the carbonation resistance of cement mortar can be effectively improved by using mixed steel slag aggregate. In particular, the carbonation depth of the B-F50 sample using 50% FSA was lower than that of the B-B50 sample using 50% BSA, indicating that the use of FSA was more effective in improving the strength and carbonation resistance of mortar than BSA.

4. By replacing 40% of the BFSP with a cement substitute, the total charge passing through each sample was approximately 5255–5568 C, which is roughly 42–45% lower than that for the control sample, and the B-F50 samples with BFSP and 50% FSA showed the maximum resistance to chloride ion penetration. In addition, in the case of the samples using the steel slag aggregate, the total charge passing through the samples using the steel slag aggregate and BFSP together was about 41.9~42.7% lower than that of the sample without the BFSP.

5. In this study, mixed use of steel slag aggregate and BFSP may reduce the early strength of the mortar, but shows excellent performance in terms of chloride-ion penetration resistance. In addition, B-F50 samples using 40% BFSP and 50% FSA showed optimal mix proportion for enhancing long-term mechanical strength, carbonation resistance, and chloride-ion penetration resistance of mortars.

Further studies are needed to establish the relationship between microstructures of cement composites and mechanical properties, durability characteristics depending on the presence of BFSP, steel slag aggregate, etc.

## Figures and Tables

**Table 1:** Chemical composition of cementitious materials.

Type	SiO_2_	Al_2_O_3_	Fe_2_O_3_	CaO	MgO	K_2_O
OPC (ordinary Portland cement)	17.43	6.50	3.57	64.40	2.55	1.17
Blast furnace slag powder	60.61	13.98	0.32	40.71	6.43	0.60

**Table 2:** Physical properties of fine aggregates.

Type	Fineness Modulus (FM)	Density (g/cm^3^)	Water Absorption Ratio (%)
Natural fine aggregate (NFA)	2.89	2.60	1.0
Blast furnace slag fine aggregate (BSA)	2.37	2.81	2.1
Ferronickel slag fine aggregate (FSA)	3.51	3.05	0.6

**Table 3:** Mix proportions.

Mix Proportion	BSA (%)	FSA (%)	W/B (%)	Water (kg/m^3^)	Cement (kg/m^3^)	Blast Furnace Slag Powder (kg/m^3^)	NFA (kg/m^3^)	BSA (kg/m^3^)	FSA (kg/m^3^)
Control C-B50 C-BFA C-F50 B-B50 B-BFA B-F50	0 50 25 0 50 25 0	0 0 25 50 0 25 50	50	170	340 340 340 340 204 204 204	0 0 0 0 136 136 136	788 394 394 394 394 394 394	0 425 212 0 425 212 0	0 0 230 460 0 230 460

## Data Availability

Not applicable.
